# Cardiometabolic Modification of Amyloid Beta in Alzheimer’s Disease Pathology

**DOI:** 10.3389/fnagi.2021.721858

**Published:** 2021-08-23

**Authors:** Marleigh Hefner, Vineet Baliga, Kailinn Amphay, Daniela Ramos, Vijay Hegde

**Affiliations:** ^1^Obesity and Metabolic Health Laboratory, Department of Nutritional Sciences, Texas Tech University, Lubbock, TX, United States; ^2^College of Arts and Sciences, University of North Carolina, Chapel Hill, Chapel Hill, NC, United States

**Keywords:** obesity, diabetes, cardiovascular disease, NAFLD, Alzheimer’s disease, amyloid precursor protein, amyloid beta

## Abstract

In recent years, several studies have suggested that cardiometabolic disorders, such as diabetes, obesity, hypertension, and dyslipidemia, share strong connections with the onset of neurodegenerative disorders such as Parkinson’s and Alzheimer’s disease (AD). However, establishing a definitive link between medical disorders with coincident pathophysiologies is difficult due to etiological heterogeneity and underlying comorbidities. For this reason, amyloid β (Aβ), a physiological peptide derived from the sequential proteolysis of amyloid precursor protein (APP), serves as a crucial link that bridges the gap between cardiometabolic and neurodegenerative disorders. Aβ normally regulates neuronal synaptic function and repair; however, the intracellular accumulation of Aβ within the brain has been observed to play a critical role in AD pathology. A portion of Aβ is believed to originate from the brain itself and can readily cross the blood-brain barrier, while the rest resides in peripheral tissues that express APP required for Aβ generation such as the liver, pancreas, kidney, spleen, skin, and lungs. Consequently, numerous organs contribute to the body pool of total circulating Aβ, which can accumulate in the brain and facilitate neurodegeneration. Although the accumulation of Aβ corresponds with the onset of neurodegenerative disorders, the direct function of periphery born Aβ in AD pathophysiology is currently unknown. This review will highlight the contributions of individual cardiometabolic diseases including cardiovascular disease (CVD), type 2 diabetes (T2D), obesity, and non-alcoholic fatty liver disease (NAFLD) in elevating concentrations of circulating Aβ within the brain, as well as discuss the comorbid association of Aβ with AD pathology.

## Introduction

Cardiometabolic disease (CMD) pathology is a complex subject due to the intersection of various metabolic, genetic, behavioral, and environmental factors (Stanhope et al., 2018). For these reasons, the rising prevalence of cardiometabolic diseases including cardiovascular disease (CVD), type 2 diabetes (T2D), obesity, and non-alcoholic fatty liver disease (NAFLD), has become a growing concern across the globe (Table 1; Bertoni et al., 2002; Thorpe and Ferraro, 2004; Perumpail et al., 2017; Taylor et al., 2017; Centers for Disease Control and Prevention, 2018; Jardim et al., 2019; Villarroel et al., 2019; Alzheimer’s Association Report, 2020; Hales et al., 2020; Mitra et al., 2020; Centers for Disease Control and Prevention, 2020). The cost of cardiometabolic diseases associated with poor diet accounts for an estimated $50.4 billion (Jardim et al., 2019) and affects 47 million (Wang et al., 2014) individuals across the United States.

**Table 1:** Alzheimer’s and cardiometabolic disease prevalence in the US, 1998 to 2020.

Disease	Prevalence (Millions)	% Total population	% Population >65 years	Mortality rate >65 years
Obesity	139^a^	42.4	42.8	19.6^f^
Type II Diabetes	34^b^	10.5	26.8	62.3^g^
Pre-Diabetes	88^b^	26.9	71.0	—
Cardiovascular Disease	85^c^	12.1	68.5	40^h^
Non-Alcoholic Fatty Liver Disease	64^d^	19.5	27.6	68.7^i^
Alzheimer’s Disease	5.8^e^	2	10	25.4^j^

^a^Hales et al. (2020)^b^Centers for Disease Control and Prevention (2020)^c^Mozaffarian et al. (2016)^d^Mitra et al. (2020)^e^Centers for Disease Control and Prevention, (2018)^f^Thorpe and Ferraro (2004)^g^Bertoni et al. (2002)^h^North and Sinclair (2012)^i^Perumpail et al. (2017) and^j^Taylor et al. (2017).

These costs are expected to dramatically rise in the future as the elderly population in developed nations continues to increase. It is estimated that by 2050, the elderly population of the United States (individuals aged >65 years) will be double that of the population of youth aged <14 years (Lau et al., 2007). The visibility of age-related neurodegenerative diseases becomes more prevalent as domestic birth rates decline in tandem with a growing elderly population, as seen by an estimated 50 million Americans displaying such symptoms each year (Brown et al., 2005). However, there is evidence to suggest the existence of an underlying relationship between deteriorating metabolic health and age-related neurodegenerative disease risk. In elderly populations, mid-life obesity, T2D, and various CVD are associated with an increased risk of dementia development (de Bruijn and Ikram, 2014), supporting the trend that the onset of age intensifies the complications and progression of neurodegenerative diseases over time.

Age-related neurodegenerative diseases, particularly dementia, progress incrementally over time. An example of this trend between age and dementia progression is observed through mild cognitive impairments, which serve as clinical precursors that characterize the stages of advanced dementias (Roberts and Knopman, 2013). Individuals diagnosed with these impairments are at an increased risk for developing dementias compared to cognitively healthy subjects, with a conversion rate ranging between 10–15% in specialty clinics (Michaud et al., 2017). It is possible for individuals with mild cognitive impairment to improve or maintain their cognitive health: however, permanent and accelerated cognitive decline is considered inevitable once an impairment proceeds toward advanced stages of dementia such as those typical to AD (Michaud et al., 2017).

Alzheimer’s disease (AD) is the most common age-related neurodegenerative disease and is the 4th leading cause of death (Estrada et al., 2019) in the developed world, accounting for 60–80% of all dementia-related cases (Alzheimer’s Association, 2008). The direct medical cost of AD-related dementias across the developed world is projected to increase from $109 billion in 2010 to $259 billion in 2040 even if the prevalence of AD were to remain constant (Deb et al., 2017). However, the sum of the economic, social, and emotional burden of AD cannot be truly quantified.

*The objective of this review* is to explore how major cardiometabolic diseases (T2D, obesity, and NAFLD) contribute to AD pathology. Due to its growing importance to medical systems, public policy, and health economics, AD will be the primary lens through which the relationship between age, metabolism, and neurodegenerative disease progression will be discussed.

## Association of The Major Cardiometabolic Diseases and Alzheimer’s Disease

### Type 2 Diabetes and Obesity

T2D is a metabolic disease of abnormal insulin function that leads to chronic hyperglycemia and impacts roughly 26 million individuals in the United States, with an estimated additional 79 million facing pre-diabetes conditions (Blair, 2016). T2D incidence is projected to increase 50% by 2040, representing a total of 640 million new cases (Zheng et al., 2018). The exponential rise in T2D cases poses significant economic and social burdens for global health systems and individuals. A crucial relationship between T2D and AD is observed in the context of chronic peripheral hyperinsulinemia and impaired insulin sensitivity being identified in patients with AD, though research on their translation to brain hyperinsulinemia and brain insulin resistance is in its infancy (Verdile et al., 2015). Promising data from fluorodeoxyglucose-positron emission tomography (also known as FDG-PET) imaging studies have observed reductions in brain glucose metabolism under conditions of hyperinsulinemia within brains afflicted with AD (Verdile et al., 2015).

Insulin signaling regulates major metabolic processes within the liver, adipose tissue, and skeletal muscle. This relationship between insulin signaling and other CMD is supported by an estimated 61% of T2D cases simultaneously correlate with obesity, therefore making obesity a strong risk factor for the development of T2D (Zheng et al., 2018). Over the last 25 years, obesity rates have risen exponentially to the extent that obesity is now classified as an epidemic affecting 1 in 3 adults in the United States (Lee, 2011). Obesity is formally defined as a metabolic disorder in which body mass index >30 kg/m^2^ (Walker and Harrison, 2015; Pugazhenthi et al., 2017) and is characterized by chronic hyperglycemia, elevated concentrations of free fatty acids in the blood *via* overnutrition, and physical inactivity (Guzman et al., 2016; Pugazhenthi et al., 2017). As lipids accumulate in adipose tissue, numerous adverse complications arise such as increases in peripheral inflammation, insulin resistance, dyslipidemia, and higher concentrations of inflammatory cytokines that play key roles in overactivation of immune response mechanisms (Lee, 2011; Verdile et al., 2015; Walker and Harrison, 2015; Pugazhenthi et al., 2017).

Obesity is also considered a catalyst for dementia, but there is an ongoing debate regarding the extent of the relationship between the two disorders. Midlife (40–59 years) has been identified as the time period with the highest risk of developing dementia for obese individuals compared to any other point in life (Walker and Harrison, 2015). The location of adipose tissue accumulation is equally as critical since abdominal adiposity is known to contribute substantially to the risk of developing insulin resistance, T2D, heart attack, heart failure, high blood pressure, and neurodegenerative diseases when compared to overall BMI (Yusuf et al., 2005; Meisinger et al., 2006; Nicklas et al., 2006; Racette et al., 2006; Yoon et al., 2012; Nurdiantami et al., 2018). Obesity is also associated with decreased hippocampal volume and a reduction of brain gray matter (Arvanitakis et al., 2004). A systematic review conducted by Albanese et al. reinforces this point by identifying that the probability of developing dementia dramatically increases as BMI increases, particularly in midlife (Albanese et al., 2017).

### Cardiovascular Disease

CVD is a large category of diseases that centers around impairment of the cardiovascular system through complications arising within heart and blood vessels that reduces functional capacity (Francula-Zaninovic and Nola, 2018). This impairment manifests in many forms ranging from heart-centric disorders such as arrythmia, valve malfunctions, heart attacks, and stroke to circulatory diseases such as coronary artery disease, deep vein thrombosis, hypertension, and atherosclerosis (Matheny et al., 2011; de Bruijn and Ikram, 2014).

CVD comprises the leading cause of death in the United States and Europe with a mortality rate of 35.2% and 48%, respectively, and is on a trajectory to average 23.6 million deaths per year globally by 2030 (Willis et al., 2012; Francula-Zaninovic and Nola, 2018). Disorders related to CVD can develop within individuals of any age; however, the risk of developing CVD rises abruptly with ages after 45 and disproportionately impacts a far greater number of individuals 65 years or older (40% mortality; Table 1, North and Sinclair, 2012). Common conditions associated with increased risks of CVD include obesity, increased blood pressure, dyslipidemia, adverse behaviors (lack of exercise, tobacco, and alcohol usage), and T2D (Willis et al., 2012). CVD does not exist in a vacuum, and individuals experiencing it are likely to develop, or be at risk of developing hypercholesterolemia, dyslipidemia, hypertension, and T2D, which increase the severity of unfavorable health outcomes (Fillit et al., 2008). With the pattern of CVD incidence strongly correlating with age, it is common to see individuals with CVD also exhibit symptoms of other age-related or neurodegenerative disorders. This is primarily due to CVD disruptions in blood circulation. These disruptions reduce oxygen and nutrient delivery to the brain as well as removal of toxic byproducts, exacerbating progressive cognitive decline associated with common neurodegenerative diseases, such as AD (Matheny et al., 2011; de Bruijn and Ikram, 2014). The risk of AD is reported to double every 5 years after age 60, and more than 30% of people over the age of 80 are likely to suffer from both AD and CVD (Tini et al., 2020).

### Non-alcoholic Fatty Liver Disease

The liver is responsible for a variety of complex metabolic processes, including macronutrient metabolism, regulation of blood volume, detoxification, immune system function, lipid and cholesterol homeostasis, and the metabolism of many drugs (Trefts et al., 2017; Estrada et al., 2019). Macronutrient metabolism is among the most critical liver functions. Additionally, the liver is involved in lipid oxidation, packaging of excess lipids for storage in adipose tissue, and ketogenesis (Trefts et al., 2017). The health of the liver is critical to wellbeing. Therefore, disruptions in the functionality of the liver through exogenous or endogenous conditions can have life-threatening consequences. One such adverse consequence is manifested in NAFLD, a disorder that results in the excessive accumulation of fat within the liver of individuals who consume little to no alcohol, the most common type of liver disease (Sheka et al., 2020). Patients experiencing NAFLD display the presence of hepatic steatosis—at least 5–10% of the liver’s weight being fat—without competing liver disease etiologies such as excess alcohol consumption or viral hepatitis (Younossi et al., 2016). Approximately 20% of individuals diagnosed with NAFLD also experience inflammation originating from increased accumulation of fat within the liver or nonalcoholic steatohepatitis (NASH; Sheka et al., 2020). Consequences of NAFLD/NASH include reduced whole-body hepatic and adipose tissue insulin sensitivity (Bugianesi et al., 2010) as well as an increased risk of cardiovascular events (Targher et al., 2020), T2D, and all-cause mortality (Targher et al., 2020). Thus, such a cascade strengthens the hypothesis that NAFLD/NASH carries a degree of influence on the incidence of other metabolic disorders such as obesity, T2D, and CVD. According to Hurjui et al., NAFLD should be considered a piece of a multi-organ system disturbance of insulin sensitivity, which explains why NAFLD episodes are closely linked with the onset of diabetes, metabolic syndrome, and CVD (Hurjui et al., 2012). Although the precise mechanism for the association between NAFLD and insulin resistance is not fully understood, current evidence indicates NAFLD alters the secretion of hepatokines, lipids, and non-coding RNA, of which the latter play a critical role in regulating gene expression (Sulaiman et al., 2019; Yoshino and Dwivedi, 2020).

As mentioned, alterations in lipid metabolism has been linked to AD risk, and due in part to the role of the liver in maintaining lipid homeostasis, the liver has been identified as a novel target for AD treatment (Stampfer, 2006; Estrada et al., 2019). In addition, researchers have identified 189 genes mutually expressed in conditions of both AD and NAFLD, and have recently linked the liver to the brain *via* the blood-brain barrier (Estrada et al., 2019). Specifically, the low-density lipoprotein receptor-related protein 1 (LRP1) is a transport protein expressed in both the blood-brain barrier and the liver, functioning to clear brain and periphery-derived amyloid β (Aβ), which is a key protein involved in AD pathogenesis. Aβ, its contribution to AD, and its relationship with metabolic health, will be discussed at length in the remainder of this review. In this review, findings from the relationship between liver dysfunction and AD will serve as the first indication that metabolic disorders, such as NAFLD, T2D, obesity, and CVD, may be a common link to AD pathology *via* Aβ modification.

## Similarities in Cardiometabolic Disease Etiology with Alzheimer’s Disease Pathology

It is important to note that the major CMD have similar risk factors, which illustrates the challenges in definitively attributing observational epidemiological studies to a single disorder. Recently, numerous studies have linked insulin resistance, a key pathophysiological feature of NAFLD, T2D, and obesity, to several neurodegenerative mechanisms of AD. These mechanisms include oxidative stress, mitochondrial dysfunction, and inflammation by dysregulated insulin signaling, along with impairments to signal transduction and gene expression (Estrada et al., 2019). Hepatic fibrosis has also been shown to lead to reduced insulin clearance, insulin resistance, and T2D (Utzschneider and Kahn, 2006). It is well established that hepatic, adipose, and muscle tissue insulin resistance and decreased pancreatic insulin production result from hyperglycemia brought about by excessive liver gluconeogenesis (Bazotte et al., 2014), which are all predictors of both T2D and NAFLD. In conditions of obesity, liver lipid accumulation has been associated with NAFLD pathophysiology (Utzschneider and Kahn, 2006); it is estimated that up to 80% of NAFLD patients are clinically obese and up to 70% of liver disease patients are overweight (Milić et al., 2014; Meex and Watt, 2017). Chronic low-grade peripheral inflammation from excess visceral adipose tissue and a surplus of circulating free fatty acids are not only two key contributors to liver damage and progression of NAFLD, but also exemplify the conditions of obesity (Milić et al., 2014). Similarly, abnormal lipid metabolism commonly found in obesogenic conditions parallels an increased risk for AD development and reinforces how dyslipidemia typical to T2D and obesity possesses significant overlap with AD. Therefore, dyslipidemia can serve as a credible risk factor that measures the metabolic irregularities that facilitate the onset of neurodegenerative disorders. This overlap of dyslipidemia between CMD and AD points to the crucial role the liver serves as the main peripheral organ responsible for lipid metabolism and indirectly links the conditions of NAFLD with AD progression (Estrada et al., 2019).

An association between NAFLD and increased CVD prevalence has also been identified (Martins and Oliveira, 2018). Much of the evidence suggests that CVD is the most common cause of mortality among patients with NAFLD (Martins and Oliveira, 2018). Mildly elevated serum gamma-glutamyltransferase concentrations independently predict the occurrence of CVD-related events and NAFLD is associated with a higher incidence of high-risk coronary atherosclerotic plaques and coronary artery inflammation (Choi et al., 2018; Wojcik-Cichy et al., 2018; Rahmani et al., 2019). The definitive contribution of NASH or NAFLD to excess CVD risk remains unanswered, but a 2010 meta-analysis by Musso et al. suggests that NAFLD patients experienced roughly twice the risk of fatal and non-fatal CVD events compared to control populations (Musso et al., 2011). Additionally, anti-aging genes might be a link between CMD and AD pathology, particularly the NAD-dependent deacetylase SIRT1. SIRT1 has protective effects against CVD and regulates blood pressure, atherosclerosis, oxidative stress and cardiac cell survival through its deacetylase activity (Tanno et al., 2012). The hallmark of AD is the accumulation of amyloid beta plaques, which result from the sequential cleavage of APP by the beta and gamma-secretases (Tanzi and Bertram, 2005). However, studies show that SIRT1 expression avoids the production of amyloid beta peptides by alternate cleavage of APP by the alpha and gamma-secretases (Postina et al., 2004; Kojro and Fahrenholz, 2005), which possible reduces brain generated amyloid beta plaques. Therefore, SIRT1 could have a therapeutic role in CMD associated neurodegenerative diseases (Martins, 2018). In summary, the human body is made up of intertwined system that cause different diseased states to have significant similarities in their presentation and etiology. Furthermore, each chronic disorder covered in this review, including obesity, T2D, NAFLD, and CVD, has been linked to AD pathology in overlapping and independent ways, which we will discuss in detail moving forward.

## The Role of Aβ in Alzheimer’s Disease

AD is a disorder characterized by a progressive, irreversible, gradual decline in cognitive function. It is neuropathologically defined by progressive neuronal and synaptic loss as well as the progressive aggregation of neurofibrillary tangles and senile plaques (Schapira, 2007). The prevalence of AD is rapidly escalating worldwide, primarily among people that exceed 65 years of age. At such rates, cases of AD incidence are projected to double by 2050 from the current 55–88 million cases globally (Hebert et al., 2001). Collectively, these data warn of the morbidity associated with AD, and the significant rise in AD cases being observed has the potential to place significant economic burden on the global healthcare apparatus.

**Figure 1:**
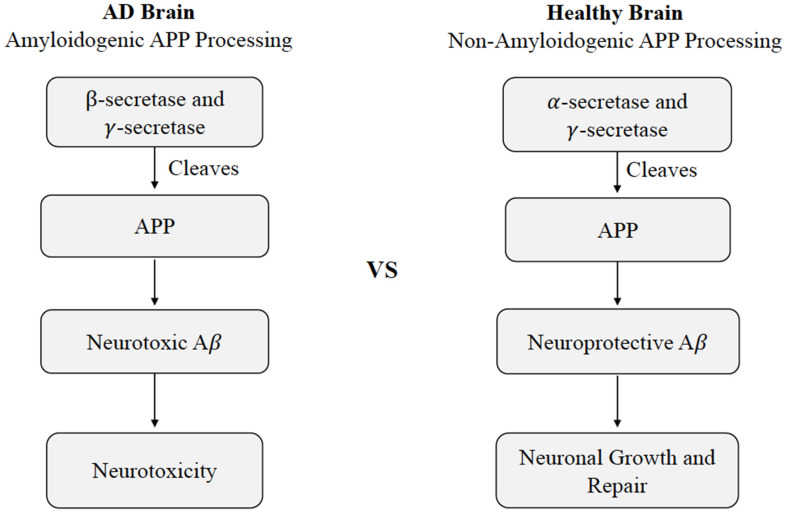
Cleavage of APP in healthy vs. Alzheimer’s disease states. Differences in non-amyloidogenic cleavage of APP in a healthy brain compared to amyloidogenic cleavage of APP AD brain. In a healthy brain, APP is cleaved by *γ*-secretase and γ-secretase to produce neuroprotective Aβ, leading to neuronal growth and repair. In an AD brain, APP is cleaved by Aβ β- and γ-secretase, leading to neurotoxicity. Abbreviations: APP, amyloid precursor protein; AD, Alzheimer’s disease; Aβ, amyloid beta.

The current understanding of AD pathogenesis is multifaceted, but this review will focus on the fundamental processes most affected by CMD health, namely extracellular Aβ deposition and intracellular tau protein hyper-accumulation. The accumulation of Aβ and tau protein are not only two key markers of AD, but also have considerable involvement with neurodegenerative mechanisms. Aβ is a major component of senile plaques and the insoluble tau protein is a major component of neurofibrillary tangles (Hebert et al., 2001). Aβ consists of 36–43 peptides that are aggregated within the large transmembrane protein, amyloid precursor protein (APP). Aβ is produced by neurons, microglia, and astrocytes in the brain and outside the blood-brain barrier by platelets, fibroblasts, osteoblasts, and skeletal muscle cells (Wang et al., 2017). Hence, a portion of Aβ is believed to originate from the brain itself, while the rest resides in peripheral tissues such as the liver, pancreas, kidney, spleen, skin, and lungs; each of these tissues express APP required for Aβ generation. Under normal conditions, APP within the brain will produce nonamyloidogenic Aβ products by α-secretase. However, amyloidogenic Aβ can accumulate as a result of enzymatic* β*-site APP cleaving enzymes BACE-1 and multi-protein γ-secretase (Figure 1; Chow et al., 2010). Defective clearance of Aβ during the cleavage of APP is understood to result in the accumulation of insoluble Aβ (Chow et al., 2010). Initially, Aβ monomers polymerize into soluble oligomers and then into larger insoluble fragments like Aβ42 that can precipitate as amyloid fibrils (Younkin, 1998). The Aβ fragments have the potential to assume a variety of different lengths, though the average length is 40 residues (Aβ 1 to 40) with a small, but considerable portion possessing lengths of 42 residues (Aβ 1 to 42; Younkin, 1998).

The neurotoxicity of Aβ is understood to originate from the extra two amino acids on Aβ 1–42 which have a higher tendency to misfold and aggregate (Ahmed et al., 2010). The Aβ monomers, which ultimately polymerize into insoluble fragments, like Aβ42, that can precipitate into amyloid fibrils (Younkin, 1998) are most commonly associated with AD (Mayeux et al., 1999). However, the amount of Aβ deposited and its distribution weakly parallels with the clinical expression of the disease, and the causal role of Aβ in AD pathology remains under debate due to accumulating evidence that supports other alternative views of etiology (Pimplikar et al., 2010; Castellani and Smith, 2011; Chételat, 2013; Moreno-Treviño et al., 2015; Tse and Herrup, 2017; de la Torre, 2018). Among these possible alternative explanations, Aβ may in fact exert its effects early on, and trigger a cascade of degenerative processes, which act independently even when Aβ is therapeutically removed (Holtzman, 2008). Additionally, Aβ is considered polymorphic, producing different conformational forms or pools of Aβ, which vary in their relevance to disease pathology (Murphy and LeVine, 2010). Even though Aβ is substantially degraded in the brain, a significant amount remains undisturbed and is transported across the blood-brain barrier into the peripheral circulation. The net concentration of Aβ transported across the blood-brain barrier is thought to determine the degree of Aβ deposited in the brain, although concrete data from human subjects is lacking (DeMattos et al., 2002). The presence of this bidirectional Aβ flow suggests that disruption of Aβ degradation in peripheral tissues by cardiometabolic impairments can contribute to the comorbid brain Aβ pathology in AD. There have been numerous studies elucidating the pathogenesis, the molecular and clinical mechanisms, the association of diabetes and metabolic syndromes, and the broad range of consequences, yet no single or combined treatment has shown to have satisfactory levels of efficiency to delay or completely prevent AD pathogenesis. Overall, AD is a complex disorder with multiple pathological contributors, some of which remain to be fully understood. As a consequence of the substantial effects that cardiometabolic modifications can have on Aβ metabolism, Aβ will be the component of AD pathology central to this review.

## Altered Aβ Metabolism in Type 2 Diabetes and Obesity

Due to their etiological similarities, T2D and obesity-induced conditions, including hyperinsulinemia, insulin resistance, hyperglycemia, altered insulin signaling, and inflammation, and their relationship with Aβ metabolism will be discussed concurrently. Other diseased states, such as NAFLD, will also be included in the following discussion but only as they contribute to such T2D and obesity-related conditions.

### Hyperinsulinemia and Insulin Resistance

As mentioned previously in this review, dysregulation of glucose homeostasis is associated with an increased risk of neurodegenerative disease development (Blázquez et al., 2014). Interestingly, in metabolically healthy individuals, it has been established that basal insulin concentration in the brain and insulin receptor affinity are significantly reduced with age (Baranowska-Bik and Bik, 2017). Therefore, one of the chief pathological features that links AD with peripheral metabolic health appears to be altered insulin metabolism (Tumminia et al., 2018). For instance, T2D is associated with impaired insulin and insulin-like growth factor-1 signaling, which has been suggested as a risk factor for cognitive impairment and dementia (Westwood et al., 2014). Hyperinsulinemia has been linked to both CMD dysfunction and AD pathology, and it highlights the close relationship between Aβ and insulin *via* insulin degrading enzyme (IDE). During hyperinsulinemia, Aβ and insulin both compete for IDE action, resulting in Aβ accumulation and plaque formation (Kawamura et al., 2012). However, isolating the individual metabolic effects of chronic hyperinsulinemia and insulin resistance is a difficult task as these phenomena tend to occur in tandem. It has been theorized that hyperinsulinemia emerged as a consequence of the insulin resistance that typifies T2D (Shanik et al., 2008), though more recent research suggests that NAFLD can serve as a significant driver of hyperinsulinemia through the pancreas even in the absence of T2D or insulin resistance (Godoy-Matos et al., 2020). In addition, it is important to note that prolonged hyperinsulinemia can result in hypoinsulinemia secondary to pancreatic beta cell fatigue.

Hyperinsulinemic conditions can also occur as a result of liver dysfunction. The excessive accumulation of fat within and around the liver induced by NAFLD can also facilitate inflammation, leading to hepatic cirrhosis and pancreatic necrosis due to their interconnected relationship (Yoon et al., 2017). For example, Yoon et al. studied 285 hospital patients diagnosed with pancreaticobiliary disease and reported that fatty liver changes matched trends associated with the development of acute pancreatitis and hepatic cirrhosis, as well as higher rates of adverse complications like mortality and organ failure (Yoon et al., 2017). A possible explanation of such conclusions is that cirrhosis induced by NAFLD can inhibit glycogenesis, which can lead to higher circulating blood glucose, bringing rise to T2D-related complications of chronic hyperinsulinemia (Yoon et al., 2017).

Furthermore, since pancreatic islet cells produce both insulin and APP, the precursor protein to Aβ, pancreatic dysfunction characterized by changes in APP concentrations is of particular interest (Yoon et al., 2017). Pancreatic dysfunction leading to altered APP cleavage and potentially neurodegenerative Aβ production has been investigated in human and animal models of AD. These studies generally suggest that increased expression of APP in the pancreas leads to increased insulin secretion in AD mice (Kulas et al., 2017). Increased concentrations of APP are among a slew of consequences of pancreatitis. On a separate but related matter, liver fat accumulation is also understood to exacerbate inflammation, contributing to the overactivation of hormonal production sites, such as the Islets of Langerhans (Yoon et al., 2017). This hormonal overactivation can lead to abnormal metabolic activity that reduces pancreatic and digestive functionality but favors overproduction of insulin from the pancreas, exacerbating conditions of increased circulating insulin that typifies chronic hyperinsulinemia (Yoon et al., 2017).

**Figure 2:**
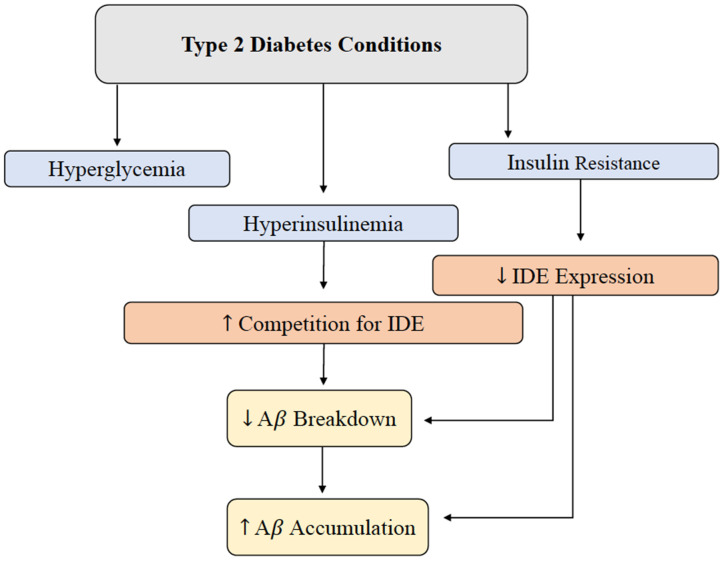
Brain Aβ accumulation induced by T2D conditions. IDE is responsible for the degradation of excess insulin and Aβ in the brain. Hyperglycemia, hyperinsulinemia, and insulin resistance are abnormal symptoms present in T2D, and this alteration in metabolism leads to a decrease in IDE expression and increased competition for IDE. Decreased IDE activity and increased IDE competition leads to decreased Aβ and insulin breakdown. In addition, hyperinsulinemia contributes to a build-up of Aβ peptides in the brain. Abbreviations: IDE, insulin-degrading enzyme; T2D, type 2 diabetes; Aβ, amyloid beta.

**Figure 3:**
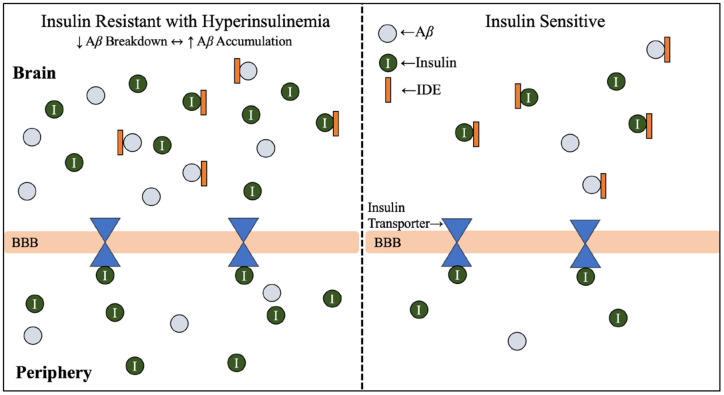
Insulin resistance and hyperinsulinemia increases competition for IDE. Mechanism by which T2D conditions of insulin resistance and hyperinsulinemia contribute to increased competition for IDE, and therefore increased accumulation of Aβ. As introduced in Figure 2, IDE is responsible for the degradation of excess insulin and Aβ in the brain. Hyperglycemia, hyperinsulinemia, and insulin resistance are symptoms of T2D, and the combined effect of hyperinsulinemia and Aβ accumulation leads to increased competition for IDE, resulting in accumulation of both Aβ and insulin in the brain. Abbreviations: Aβ, amyloid beta; AD, Alzheimer’s disease; IDE, insulin degrading enzyme; T2D, type 2 diabetes; BBB, blood-brain barrier.

Hyperinsulinemia and its relationship with AD will be further investigated throughout this review, but this relationship is introduced in Figure 2 (Qiu and Folstein, 2006). As hyperinsulinemic conditions prevail, the demand placed on the body and brain to clear insulin increases. Therefore, there is increased competition for IDE in the brain and peripheral tissues. IDE is an ubiquitously expressed enzyme dually equipped to degrade not only excess insulin, but also excess Aβ (Pivovarova et al., 2016). When IDE is occupied degrading excess insulin in conditions of hyperinsulinemia, Aβ is left to steadily accumulate in both peripheral circulation and centrally, intensifying the conditions of AD induced by Aβ accumulation (Figure 3). It is this mechanism that highlights the interconnected relationship between T2D conditions and AD pathology. Common trends between hyperglycemia, brain insulin resistance, and improper function of the IDE have been recognized in a variety of studies tracking the pathological features of AD (Tumminia et al., 2018).

Lastly, cerebrospinal insulin concentrations are correlated with peripheral insulin concentrations, suggesting pancreatic insulin makes up the majority of brain insulin (Arnold et al., 2018) and strengthens emphasis on peripheral organ system health in AD prevention and treatment. It is important to note that the ratio of the concentration of insulin in cerebrospinal fluid compared to the periphery is lower in both aging patients with AD and those with insulin resistance compared to healthy subjects. This relationship may be related to decreased transport of insulin across the blood-brain barrier since insulin transport is affected by inflammation, obesity, hyperglycemia, and hypertriglyceridemia (Banks et al., 2012). However, the ratio of cerebrospinal fluid to peripheral insulin may not be as crucial as the total concentration of insulin in the body.

### Hyperglycemia and Altered Insulin Signaling

It has also been theorized that chronic hyperglycemia may contribute to the production of advanced glycation end products (AGE), which interact with specialized AGE receptors on the endothelial lining of the blood-brain barrier that mediate Aβ influx to the brain (Li et al., 2019). Aβ is considered an AGE receptor ligand and therefore can effectively bind to these receptors and signal an influx across the blood-brain barrier from the circulatory pathways (Figure 5; Murphy and LeVine, 2010; Li et al., 2019).

**Figure 4:**
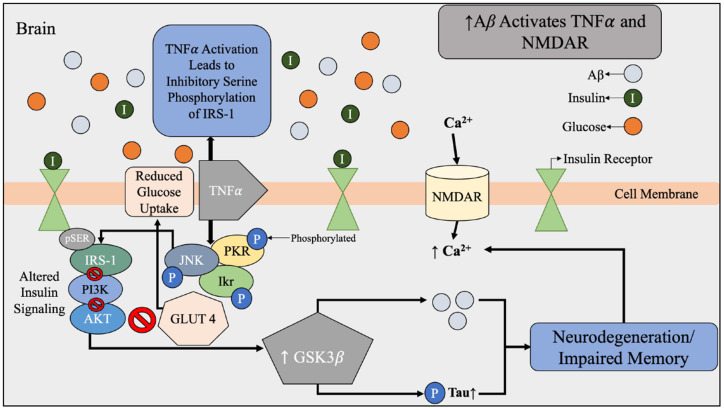
Aβ dysfunction contributing to alterations in brain insulin signaling beginning with the activation of TNFα and NMDAR by Aβ. During Aβ accumulation found in AD brains, there is increased expression of TNFα and activation of NMDAR. NMDAR activation results in an influx of Ca^2+^ that leads to oxidative stress and synaptic dysfunction of the brain. Under normal conditions, Aβ is used to regulate the calcium flow that is required for synaptic transmission. Increased expression of TNFα and stress kinases leads to inhibitory serine phosphorylation of IRS-1 and PI3K therefore reduced glucose uptake. The effects of Aβ accumulation and toxic stress triggers a cascade of impaired insulin signaling. Abbreviations: Aβ, amyloid beta; TNFα, tumor necrosis factor α; NMDAR, N-Methyl-D aspartate receptor; IRS-1, insulin receptor substrate-1; PI3k, phosphatidylinositol-3-kinase; AKT, protein kinase B; pTau, hyperphosphorylated tau; GLUT4, glucose transporter 4; JNK, N-terminal kinases; PKR, protein kinase R; Ikr, rapid-component delayed-rectifier potassium channel.

**Figure 5:**
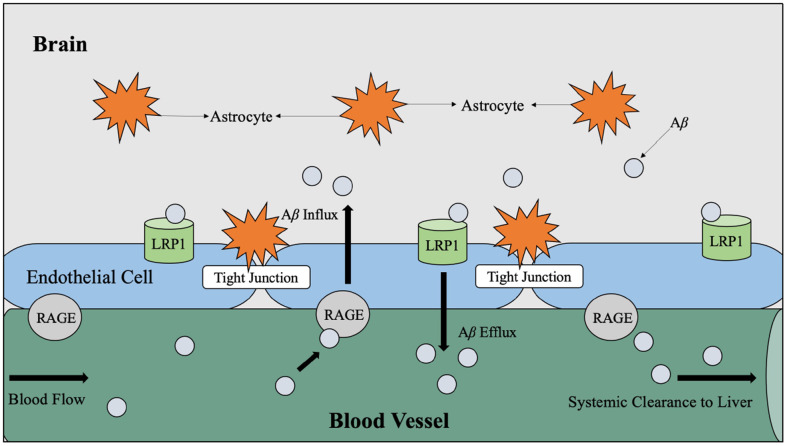
LRP1 and RAGE-mediated Aβ transport across the BBB. Aβ travels through the blood vessels to cross the BBB for systemic clearance by the liver. In the periphery, Aβ binds to RAGE, which is the receptor for AGE believed to be produced as a result of hyperglycemia. When Aβ binds to RAGE, Aβ influxes into the brain *via* endothelial cells. Counteracting Aβ influx into the brain, Aβ efflux into the periphery is mediated by LRP1. Abbreviations: LRP1, lipoprotein receptor-related protein; RAGE, receptor for advanced glycation end products; Aβ, amyloid beta; BBB, blood-brain barrier; AGE, advanced glycation end products.

Another dimension of the relationship between insulin and Aβ includes genetic and structural disruptions within the insulin signaling pathway *via* receptor-level inhibition. As concentrations of insulin increase, more insulin receptor sites are occupied; however, chronic exposure to insulin reduces the proportion of short, higher-affinity isoforms of insulin receptors relative to the long, lower-affinity isoforms (Shanik et al., 2008). In addition, serine rather than tyrosine phosphorylation of the insulin response element has been associated with insulin resistance related to decreased activation of downstream insulin-signaling proteins (Figure 4; Copps and White, 2012). This alteration of insulin signal pathways intricately links peripheral insulin resistance to brain insulin resistance, in addition to the discovery that insulin receptors are widely expressed in the brain (Pomytkin et al., 2018). Brain insulin and glucose metabolism play an important role in synaptic function of neurons (Figure 4) and Aβ degradation (Figure 3), in addition to their chief role of glucose uptake (Freude et al., 2005; De Felice et al., 2014; Bedse et al., 2015; Arnold et al., 2018). In summary, glucose metabolism dysfunction in the form of hyperinsulinemia, insulin resistance, hyperglycemia, and altered insulin signaling are all metabolic factors that can be individually and holistically linked to Aβ metabolism, and therefore AD risk.

### Inflammation

**Figure 6:**
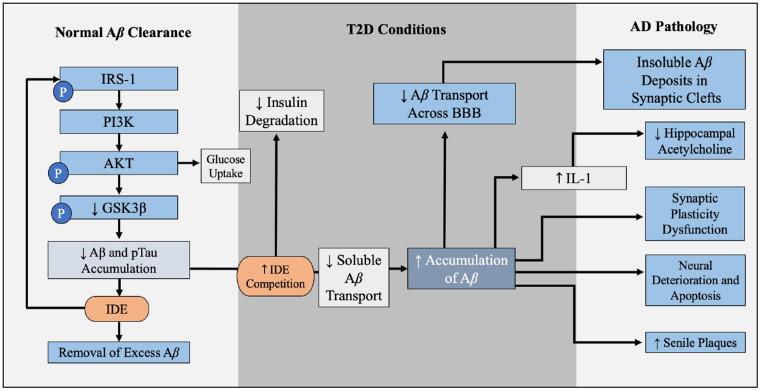
Contribution of T2D conditions to Aβ accumulation and AD pathology. In the healthy brain insulin signaling pathway, insulin binds to PI3K which is degraded to AKT, leading to GLUT4-mediated glucose uptake and phosphorylation (inactivation) of GSK3β. GSK3β inactivation leads to decreased Aβ and pTau accumulation, and IDE degrades excess insulin and Aβ in the brain. When IR and hyperinsulinemia is developed during T2D, this increased competition for IDE, leading to Aβ and insulin accumulation, further illustrated in Figure 4. Increased Aβ accumulation leads to increased decreased soluble Aβ transport across BBB, leaving insoluble Aβ deposits trapped within the synaptic clefts, promoting inflammation and oxidative stress and increasing NF-kB. Increased levels of NF-kB increases IL-1 expression, which reduces hippocampal acetylcholine. Excess Aβ decreases synaptic plasticity, promotes neural deterioration and apoptosis, and promotes Aβ plaques. Aβ plaques is a well-established contributors to AD. Abbreviations: Aβ, amyloid beta; T2D, type 2 diabetes; AD, Alzheimer’s disease; PI3K, phosphoinositide 3-kinase; AKT, protein kinase B; GLUT4, glucose transporter 4; GSK3β, glycogen synthase kinase 3 β; pTau, hyperphosphorylated tau; IDE, insulin degrading enzyme; IR, insulin resistance; BBB, blood-brain barrier.

Chronic inflammation is prevalent in multiple metabolic diseases, including T2D and obesity, and may serve as another possible risk factor in appraising AD risk (Murphy and LeVine, 2010). Increased concentrations of pro-inflammatory cytokines produced in the presence of chronic inflammation can lead to pancreatic β-cell damage and impaired insulin secretion (Murphy and LeVine, 2010). These cytokines, such as tumor necrosis factor-α (TNF-α), have the ability to cross the blood-brain barrier to contribute to the progression of AD (Figure 4; Wang et al., 2015). In addition, it has been established *in vivo* since the 1990’s that when the interleukin-1 is elevated in the brain, less hippocampal acetylcholine is produced, and this can contribute to neurological deficits (Rada et al., 1991; Figure 6).

Oxidative stress is understood to begin with the production of reactive oxygen species, which are molecules that contain oxygen free radicals and are formed during normal cellular processes (de la Monte, 2012). This production of free radicals contributes to oxidative stress and cellular damage. Due to the brain’s elevated lipid concentration, lack of antioxidant defense mechanisms, and high oxygen requirements make the brain especially vulnerable to oxidative stress, which is a well-established early contributor to AD-related neurodegeneration (Verdile et al., 2015). Furthermore, inflammation-induced oxidative stress in the brain can lead to altered brain glucose metabolism and reduced ATP synthesis, which promotes neural dysfunction, synapse loss, and neurodegeneration (Verdile et al., 2015). *In vitro* evidence also suggests that Aβ may increase the generation of reactive oxygen species; however, it is unclear whether Aβ are the cause or result of oxidative stress when *in vivo* evidence is presented (Yao et al., 2009). Furthermore, the primary site for reactive oxygen species generation is the mitochondria, and mitochondrial dysfunction has been suggested as an early contributor to AD, particularly in mouse models (Verdile et al., 2015). Chronic inflammation is an important component of altered Aβ metabolism through the lens of obesity as well.

In a corresponding manner, adipose tissues release adipokines, such as leptin and adiponectin that function in a similar manner to that of cytokines with regards to oxidative stress and inflammation (Verdile et al., 2015). Interestingly, leptin, the primary regulator of appetite and energy homeostasis is related to insulin resistance and the development of obesity when produced in excess (Gruzdeva et al., 2019). As tissues become less sensitive to leptin, a condition known as leptin resistance, the failure of leptin feedback mechanisms lead to continued production by adipose tissues despite the already synthesized leptin being unable to carry out its function or participate in signaling cascades. Leptin resistance and excess leptin production result in abnormal appetite, hyperinsulinemia, hypothalamic inflammation, obesity, dyslipidemia, altered blood-brain barrier transport, and the emergence of other metabolic disorders (Gruzdeva et al., 2019). Leptin can cross the blood-brain barrier to evoke the classical appetite-suppressing and thermogenesis-initiating signals to the hypothalamus (Banks, 2001). Increased concentrations of circulating leptin in obesity are known to increase neuroendocrine dysfunction exacerbated by hypothalamic leptin resistance, which leads to the down regulation of appetite-suppressing and energy-expending signals, contributing to obesogenic conditions (Waterson and Horvath, 2015).

Although leptin receptors are heavily concentrated within the hypothalamus, other regions of the brain associated with learning and memory, such as the hippocampus, express leptin receptors as well, which explains why these regions are widely focused on in AD and obesity-related research (Murphy and LeVine, 2010). In fact, direct injection of leptin within the hippocampus of mice has been shown to modulate long-term nerve impulse strength and synaptic plasticity, as well as dramatically improved memory processing in both time-dependent and dose-dependent conditions (Li et al., 2016). In human studies, decreased leptin levels is associated with increased Aβ deposition and memory impairment, reinforcing how leptin resistance within the brain may exacerbate AD-related pathologies (Li et al., 2016; Forny-Germano et al., 2018). Collectively, the evidence suggests leptin may play a role in neuroprotection and the effects of obesity on neurodegenerative disorders.

Beyond chronic inflammation induced by inflammatory adipokines and cytokines, the current understanding of altered Aβ metabolism in the context of obesity is related to T2D due to the similarities in the pathophysiology of each condition. Most relevant to dysfunctional Aβ metabolism is the mutual presence of insulin resistance in conditions of obesity and T2D. Therefore, the mechanism of altered Aβ metabolism in the context of insulin resistance is identical for both T2D and obesity. Obesity is unique in its contribution to insulin resistance compared to T2D because excess adiposity is understood to result in insulin resistance, whereas T2D conditions often begin with insulin resistance. Nevertheless, there is a considerable lack of evidence that excess adiposity alone may contribute to Aβ metabolism.

## Altered Aβ Metabolism in Cardiovascular Disease

CVD pathology can be summarized as elevated levels of blood lipids paired with atherosclerosis, the buildup of lipids, cholesterols, and plaques within arterial walls, that can lead to arterial stiffness and rigidity and obstruct blood flow and increase blood pressure (de Bruijn and Ikram, 2014). Elevated blood pressure damages arteries and other blood vessels which can lead to chronic inflammation (Fioranelli et al., 2018). In addition, chronic inflammation drastically reduces the functional surface area of blood vessels due to their inability to completely repair. Reduced functional surface area can lead to the deposition and accumulation of proteins like pTau and Aβ secondary to microglial cells lacking the adequate oxygen and glucose to perform routine excretory functions at maximum efficiency (Fioranelli et al., 2018). As cardiovascular circulatory efficiency diminishes, brain atrophy can increase, which is understood to be related to inefficient Aβ clearance and removal (Murphy and LeVine, 2010). Furthermore, brain Aβ concentrations are proportional to cholesterol concentrations, and Aβ accumulation within heart and blood vessels is understood to contribute to arterial stiffening, atherosclerosis, and cardiac dysfunction (Yeung et al., 2014; Fioranelli et al., 2018). In addition, Aβ1–40 was found to be 100 times more abundant in aortic atherosclerotic plaques than anywhere else in the body (Yeung et al., 2014). This accumulation of Aβ occurs when lymphatic capillaries fail to adequately drain due to the atherosclerosis, contributing to greater Aβ1–40 deposits within cardiovascular structures that induce cell damage and inflammation (Yeung et al., 2014).

A New York Heart Association study of 939 patients exhibiting early signs of heart failure indicated that higher concentrations of circulating Aβ1–40 resulted in higher mortality rates and greater frequency of diastolic dysfunction (Stakos et al., 2020). These adverse outcomes are likely due to elevated Aβ1–40 hindering the blood supply by disrupting normal vascular development. As the blood supply decreases, APP is upregulated, leading to increased vasoconstriction that inhibits the functionality of endothelium that is necessary in maintaining adequate blood supply to critical organs by vasodilation (Stakos et al., 2020). A similar experiment exploring the relationship between Aβ and diastolic dysfunction involving 3,266 patients discovered that lower stroke volume corresponds with higher concentrations of Aβ, reinforcing the results of the first (Stakos et al., 2020).

Furthermore, knockout mice for the APP gene (APP−/−) displayed 75–90% smaller arterial plaque thickness, significantly fewer arterial lesions, and a lower pro-inflammatory response measured by a low macrophage content than the APP+/+ mice (Karisetty et al., 2020; Tini et al., 2020). Therefore, APP, and subsequently Aβ, may play a role in the vascular inflammation that exacerbates cognitive impairment (Stakos et al., 2020). Although the aforementioned studies do shed light on the relationship between CVD conditions and Aβ metabolism independent of neurodegenerative outcomes, additional research is required to determine a direct relationship between CVD pathology and AD risk.

## Altered Aβ Metabolism in Non-Alcoholic Fatty Liver Disease

The liver has been identified as a key organ involved in the pathology and progression of AD due to its role in Aβ clearance (Estrada et al., 2019). It has been observed that Aβ solubility, as well as functionality of Aβ transport proteins, is of great importance with regards to transport across the blood-brain barrier when the liver enters a diseased state. In addition, evidence suggests that the solubility of Aβ is a critical aspect of clinical manifestations of AD (Murphy and LeVine, 2010).

The main enzyme responsible for Aβ production is BACE1, which is primarily expressed in the brain and can be found in peripheral organs (Murphy and LeVine, 2010). Under normal physiological conditions, Aβ is produced to support synaptic plasticity and regulate the calcium flow required for the transmission of synaptic communication(Karisetty et al., 2020). In the periphery, Aβ clearance is an essential contributor to AD progression since inadequate removal of Aβ from blood contributes to Aβ accumulation in the brain (Wang et al., 2006). Although enzymes such as IDE and neprilysin are responsible for degrading excess Aβ within the brain (Figure 3), much of the Aβ remains intact. Therefore, the efflux of soluble Aβ across the blood-brain barrier to the periphery exists as a mechanism for enhanced Aβ clearance (Murphy and LeVine, 2010).

As introduced in Figure 5, it is hypothesized that the facilitation of soluble Aβ efflux across the blood-brain barrier is controlled by the low-density lipoprotein receptor-related protein 1 (LRP1) on the “brain side” and receptors for advanced glycation end products (RAGE) on the “blood side” (Murphy and LeVine, 2010). The LRP1 is understood to be the mediator for Aβ uptake by hepatocytes (Wang et al., 2017). Theoretically, the proper flow of Aβ across the blood-brain barrier for clearance by the liver reduces Aβ burden on the brain since it is estimated that 60% (Wang et al., 2017) of brain-derived Aβ is cleared by the periphery; however, insoluble Aβ is a characteristic of AD pathology and cannot cross the blood-brain barrier (Murphy and LeVine, 2010). Therefore, soluble Aβ is considered more toxic than insoluble Aβ (Li et al., 2019).

In the periphery, soluble Aβ clearance is an essential component to AD progression since inadequate removal of Aβ from blood circulation contributes to Aβ buildup in the brain (Wang et al., 2006). Impaired removal of Aβ has been proposed as a potential link between peripheral metabolic dysfunction and AD; therefore, peripheral organs may be a vital component of excess Aβ removal. The liver, in particular, is unique in that it promotes Aβ clearance by protein degradation or bile excretion (Estrada et al., 2019). Liver disease is associated with high concentrations of circulating Aβ as a consequence of diminished detoxification ability (Wang et al., 2017). To counteract this effect, the administration of treatments that enhance LRP1-mediated Aβ clearance by the liver result in both improved cognition and mitigate the potentially damaging effects of Aβ within the brain (Tamaki et al., 2007; Sehgal et al., 2012). Hence, therapeutic intervention focused on the liver emerges as a promising target for AD intervention and therapy.

## Ad Treatments and Future Directions

### Currently Available Alzheimer’s Disease Treatments

At present, there are only two drug classifications for AD treatment that are clinically proven to improve memory and alertness, but do not increase life expectancy or slow AD progression. These drugs are cholinesterase inhibitors and memantine (Table 2, Weller and Budson, 2018). Cholinesterase inhibitors (donepezil, rivastigmine, and galantamine) are prescribed for the specific treatment of various stages of AD, whereas memantine is reserved for moderate or severe cases of AD (Weller and Budson, 2018). Memantine medications have been experimentally shown to over stimulate the extra synaptic N-methyl-D-aspartate receptor (NMDA), which is understood to contribute to neuronal excitotoxicity and death (Wang and Reddy, 2017), however, clinically, the effect of memantine appears to be more of an antagonistic effect on the whole population of NMDA receptors (Liu et al., 2019).

**Table 2:** Approved Alzheimer’s disease treatments.

Reference	Drug class	Mechanism	Key findings
Sharma (2019)	Cholinesterase inhibitors	Blocks cholinesterase, which degrades acetylcholine.	Limited efficacy, high doses associated with negative side effects, such as worsened cognition.
Matsunaga et al. (2015)	Memantine	Blocks NMDA receptors, preventing neuron loss.	More promising efficacy, some improvements in cognition, well tolerated, but small effect sizes of clinical studies limit evidence.

*Abbreviation: NMDA, N-Methyl-D-aspartic acid or N-Methyl-D-aspartate*.

Though the purpose of the following section of this review is not to systematically synthesize all approved and pending AD treatments, it is critical to have a general understanding of the AD therapy-related research being conducted. The nature of this review focuses on the amyloid hypothesis, which hypothesizes that the accumulation of Aβ within brain parenchyma drives synaptic dysfunction and neurodegeneration leading to the manifestation of AD (van Dyck, 2018); however, both anti-amyloid and non-anti-amyloid treatments under investigation have been included as this review emphasizes the potential for combination therapies (Table 3) for effective AD treatment. Aβ is considered the initiator of the AD cascade, beginning with Aβ-induced amyloid plaques, neurofibrillary tangles from pTau, neurodegeneration, synaptic damage, neuron loss, and other pathological hallmarks of AD (Chen et al., 2017). As a result, anti-amyloid therapies have an extensive history of intervention attempts. AD patients have also displayed deficiency in the enzyme choline acetyltransferase, hence, cholinergic-based therapies were created (Sharma, 2019). Acetylcholine plays a sizable role in learning and memory, which is why it is now classified as a key biomarker in late-term AD pathology.

**Table 3:** Major anti-amyloid AD treatments under investigation.

Reference	Drug class	Mechanism	Key findings
Moussa-Pacha et al. (2020)	BACE1	BACE inhibitor, reduces Aβ production.	Insignificant physiological effects, some shown to worsen cognition. Several early phase trials ongoing.
Hung and Fu (2017)	pTau	Derivative of methyl blue shown to inhibit tau aggregation.	Phase 2 clinical trial suggests improvement in cognition at low doses. Research ongoing.
Hung and Fu (2017)	pTau	Enhancing immunotherapy pTau clearance *via* synthetic peptide of truncated and misfolded tau.	All early phase trials. No data yet but research ongoing.
Hung and Fu (2017)	pTau	Microtubule stabilizing agents.	Several failed due to toxic side effects. Additional clinical trials in early phase.
Boada et al. (2020)	Albumin 1 Immunoglobulin Plasma Exchange	Removal of Aβ bound to albumin plasma.	New therapy. Suggests greater effect on cognition in moderate stages of AD, additional research needed.
Deane (2012)	RAGE	Antagonist of RAGE receptors transporting circulating Aβ to brain.	Clinical trials not effective.
van Dyck (2018)	Aβ	Human monoclonal anti-Aβ antibodies provide passive immunity against Aβ accumulation.	Completed trials have yielded either failed or seen non-significant outcomes. Aducanumab specifically shows more promise for improving MMSE and CDR but is in early phase testing.
Hung and Fu (2017)	Aβ	Aβ clearance enhanced by active immunotherapy from Aβ peptides.	Several trials discontinued due to adverse immune responses. Additional trials ongoing in early phases.
Hung and Fu (2017)	γ-secretase	γ-secretase inhibitors shift APP cleavage toward production of shorter, less toxic Aβ peptides.	Clinical trials discontinued due to adverse side effects. One small unpublished study suggested some improvement in cognition.
Hung and Fu (2017)	Intravenous Immunoglobin	A naturally occurring antibody from the plasma of healthy donors. IVIG may direct antibodies against Aβ.	No beneficial effects observed.
Avgerinos et al. (2018)	Intranasal Insulin	Brains affected by AD show decreased concentration of insulin and increased concentration of insulin receptors, modulated Aβ in early AD.	Some positive results in function status and ADL but no other cognitive indicators. Has been suggested that different insulin types and doses may have variable effects on different APOE patients.

*Abbreviations: BACE1, β-secretase 1; pTau, hyperphosphorylated tau; Aβ, amyloid beta; AD, Alzheimer’s disease; RAGE, receptor for advanced glycation end products; MMSE, Mini Mental State Examination; CDR, Clinical Dementia Rating; APP, Amyloid Precursor Protein; APOE, Apolipoprotein E; APOE ɛ4 allele is a risk factor for AD*.

### Alzheimer’s Disease Treatments Under Investigation

Since 2019, AD drug intervention research seems to have shifted away from anti-amyloid therapies. In addition to the major non-anti-amyloid therapies (Table 4), new experimental treatments and therapies are focusing on inducing ketosis, partial agonists of dopamine, microglial activation inhibitors, RAGE antagonists, bacterial protease inhibitors, serotonin uptake inhibitors, γ-secretase inhibitors, antioxidant and anti-neuroinflammatory agents, anti-diabetes medications, tyrosine kinase inhibitors, and PPAR-γ agonists (Huang et al., 2020).

**Table 4:** Major non-anti-amyloid AD treatments under investigation.

Reference	Drug target	Mechanism	Brief conclusions
Fitz et al. (2019)	Retinoid X Receptors	Important for APOE expression, retinoid acid-mediated signaling, neuronal plasticity, and memory; anti-inflammatory effects of RXR/LXR.	Research is focused on activating LXR/RXR in the brain using nonsteroid synthetic ligands. Mice studies have seen variable outcomes.
Fitz et al. (2019)	Liver X Receptors	Include LRXα and LRXβ. LRXs are transcriptional regulators of lipid metabolism and inflammation. LRXβ is expressed in brain and spinal cord as the brain is the most cholesterol-rich organ and peripheral cholesterol cannot cross BBB. LRXβ K/O mice display motor neuron degeneration and impaired coordination.	Early phases of research; still needs validation in animal studies but has shown reduced levels of insoluble Aβ in mice. Research is focused on activating LXR/RXR in the brain using nonsteroid synthetic ligands.
Fitz et al. (2019)	Nuclear Receptors	Widely expressed in the brain. By directly binding to PPARs and LXRs, nuclear receptors ultimately induce stabilization of nuclear complexes that promote neuro- and peripheral inflammatory genes.	Early phases of research; still needs validation in animal studies.

*Abbreviations: RXR, retinoid X receptors; APOE, apolipoprotein E; LXR, liver X receptors; BBB, blood-brain-barrier; K/O, knock-out; PPAR, peroxisome proliferator-activated receptor*.

Additional AD hypotheses, including the cholinergic hypothesis and tau hypothesis, have been targeted for anti-AD treatments. The latter can be summarized by pTau causing an accumulation of neurofibrillary tangles within nerve cells, which interact with cellular proteins and prevent them from executing their normal function (Maccioni et al., 2010; Bakota and Brandt, 2016). The cholinergic hypothesis can be summarized as a reduction in acetylcholine synthesis, which inhibits biological activity of acetylcholinesterase (Sharma, 2019). A loss of cholinergic neurons are thought to encourage excessive influx of calcium into cells, resulting in neuronal damage (Sharma, 2019). Hence, acetylcholinesterase inhibitors are utilized for treatment to limit acetylcholine degradation, increasing the function of neural cells by increasing the concentration of acetylcholine (Sharma, 2019). Still, focusing on Aβ, pTau, or acetylcholine alone is unlikely in addressing the complete range of symptoms associated with neurological disease pathology (Fessel, 2018).

Overall, Aβ therapies have not yielded promising results in clinical trials (Panza et al., 2019a). Several anti-amyloid drug therapies since 2016 have failed due to lack of efficacy, toxicity, and worsening cognition (Huang et al., 2020). There is, however, evidence that Aβ and tau pathology, currently accepted as trademark biomarkers for AD, are only a piece of the full pathogenesis of AD (Bednar, 2019). Therefore, the greatest potential for a complete and robust AD treatment lies in combination therapies individualized to each patient care based on their specific clinical manifestations of AD and from learning more about the mechanisms of pathogenesis, which can vary greatly between patients (Bednar, 2019). Instead, the barrier that AD researchers face may not be that AD treatment candidates are entirely unsuccessful, but that they are simply only addressing one aspect of an extremely complex disorder that possesses numerous overlaps with other non-neurological disorders like NAFLD and T2D that lack thoroughly understood links to AD.

### Anti-diabetes Medications for Alzheimer’s Disease Treatment

Anti-diabetes drug therapies have been investigated for their efficacy as AD therapies due to the current understanding of the mechanisms that closely link glucose metabolism dysfunction with AD (Boccardi et al., 2019). It remains unclear which anti-diabetic medications holistically improves the outcome of AD patients; therefore, it is important to identify which diabetes medications lead to improvement of cognitive function and whether these benefits supersede risk factors.

According to a systematic review of randomized controlled trials by Avgerinos et al., intranasal insulin delivers insulin to the brain more rapidly than subcutaneous or intravenous insulin due to its unique ability to cross the blood-brain barrier independent of a transport process (Avgerinos et al., 2018). Intranasal insulin in patients without existing hyperinsulinemia, has been shown to alleviate the symptoms that link AD and T2D, including Aβ accumulation, synaptotoxicity, and oxidative stress (Avgerinos et al., 2018). Overall, increasing insulin in the brain of hypoinsulinemic patients translates to decreased Aβ-induced memory loss, reduces neuronal apoptosis, and increases synaptic plasticity (Boccardi et al., 2019). Data from the same systematic review also discovered that patients with the APOE4(+) gene remained stable or declined cognitively after intranasal insulin was administered, while those with the APOE4(-) gene showed more cognitive gains after administration (Avgerinos et al., 2018). However, additional research is needed to determine whether intranasal insulin alone is a sufficient treatment for AD.

Thiazolidinediones (TZDs) containing dipeptidyl peptidase-4 (DPP-4) inhibitors and glucagon-like peptide 1 (GLP-1) receptor agonists have been shown to counteract hyperglycemia, insulin resistance, and a variety of oxidative stressors, which are all precursor metabolic processes that contribute to Aβ and pTau accumulation (Boccardi et al., 2019). The peripheral effects of these antihyperglycemic medications include improving insulin signaling and reducing inflammation and oxidative stress (Boccardi et al., 2019). Centrally, these medications increase neuroprotection, neurogenesis, and synaptic plasticity and alleviate neuroinflammation (Boccardi et al., 2019). In addition, thiazolidinediones are insulin sensitizers and agonists of the peroxisome proliferator-activated receptor gamma (PPARγ; Boccardi et al., 2019). PPARγ is a possible mechanism for AD treatment due to existing evidence that PPARγ agonists decrease Aβ accumulation and inflammation (Avgerinos et al., 2018). In addition, PPARγ agonists play a key role in regulating calcium metabolism in the hippocampus, resulting in decreased oxidative stress and other stress-activated protein kinases that contribute to insulin resistance in the brain (Avgerinos et al., 2018). TZDs have also been shown to improve cerebral blood flow *via* anti-inflammatory and insulin-sensitizing effects, which translated to increased memory in AD patients (Boccardi et al., 2019). TZDs also inactivate GSK3γ and decrease tau production (Figure 4). They do not prevent AD *per se*, but seem to slow AD progression (Boccardi et al., 2019). Like thiazolidinediones, GLP-1 receptor agonists are a group of anti-diabetic medications that have anti-hyperglycemic effects which may have beneficial effects on AD progression. GLP-1 is a short-acting insulinotropic peptide that enhances insulin gene expression while suppressing glucagon secretion in the pancreas (Boccardi et al., 2019). In *in vivo* models of AD, injection of GLP-1 receptor agonist was shown to decrease overburden of the hippocampus and improve spatial memory, and in a mouse model of T2D mice, GLP-1 injection decreased Aβ plaque in the cortex, restored peripheral and brain insulin sensitivity, and improved tau hyperphosphorylation, which are all significant features of AD (Boccardi et al., 2019).

It is important to note that healthy lifestyle choices can amplify the effects of anti-diabetic medication effectiveness. For patients suffering from hyperinsulinemia due to T2D and competition for IDE from Aβ, exercise can function as an alternative in regulating blood insulin and glucose levels as skeletal muscles possess a non-insulin-dependent ability to absorb glucose when their glycogen stores deplete post-exercise. This peripheral glucose uptake and insulin utilization has the potential to reduce hyperinsulinemia and decrease the competition for IDE discussed extensively in this review (Figure 3).

### Potential Future Directions for Alzheimer’s Disease Treatments

Neuroinflammation in response to glial cell accumulation has been recently considered as another key factor in AD pathology since amyloid plaques and neurofibrillary tangles are understood to be possible catalysts for the inflammatory response in the brain (Briggs et al., 2016). Meta analyses have suggested steroidal and non-steroidal anti-inflammatory drugs induce negligible effects on AD in symptomatic patients, but perhaps show potential for early-stage prevention of the disease (Briggs et al., 2016). Immune-based interventions, however, are predicted to lead to future preventive or therapeutic interventions for AD (Ozben and Ozben, 2019).

Stem cell therapies have been tested but have not yielded clinical efficacy yet, but more sophisticated studies are currently underway (Kang et al., 2016). Similarly, antibacterial therapy is considered another link to AD in which gut dysbiosis and neuroinflammation from the periphery exacerbate increased permeability of the gut and the blood-brain barrier (Panza et al., 2019b). At present, researchers have not finalized the safety of antibacterial therapy to AD as it is currently unknown which specific bacterial population in the gut of patients diagnosed with AD are overrepresented (Panza et al., 2019b).

In summary, there seems to be rationale for further research involving neuroinflammation and anti-diabetes-based interventions in the context of combination therapy, though it is impossible to rule out Aβ, pTau, and cholinergic focused interventions that have been tested only in solidarity. Until AD treatment can be identified, the importance of metabolic health, especially liver and glucose metabolism health, to prevent peripheral contribution of Aβ and pTau (Bharadwaj et al., 2017; Maarouf et al., 2018; Xin et al., 2018) should be emphasized by healthcare professionals (Figure 6). Research efforts for the development of AD interventions to be utilized in combination therapies may best be spent targeting Aβ clearance by decreasing LRP-1 transport of Aβ to the brain, improving Aβ clearance *via* the liver, or increasing IDE’s affinity for Aβ in the brain. In addition, low-risk preventative measures, such as eating a balanced and nutrient-dense diet, engaging in regular aerobic exercise, and supplementation for nutrient deficiencies are recommended (Mendiola-Precoma et al., 2016). As nutrition scientists know, nutrition research is challenging and often puzzling as humans are subject to behavioral, environmental, and social factors, which can rarely be accurately or reliably predicted (Bednar, 2019).

## Conclusion

The relationship between cardiometabolic health and AD pathologies are dynamic and interwoven. Aβ, one of the major pathologies of AD, served as the focus of this review. The central theme of this review was the unique relationship between Aβ and cardiometabolic health. Namely, we assessed not only how Aβ can alter cardiometabolic health, but also how cardiometabolic health can modify Aβ metabolism. Collectively, it appears insulin and inflammation are the two features common to all major CMDs and altered Aβ metabolism. This pattern reinforcing the importance of monitoring peripheral organ health in preventative neurological treatments and interventions. Based off these observations, there is reasonable evidence to suggest a collective association between AD and cardiometabolic health.

Potential novel mechanisms for AD therapies in patients with peripheral hyperinsulinemia include reducing the load of insulin on IDE in the brain, either by increasing the affinity of IDE for Aβ or by decreasing brain insulin concentrations. Furthermore, due to its central role in both Aβ clearance and hormonal regulation, the liver should be considered as a focal point of future cardiometabolic treatments targeting AD. To be effective, these therapies should improve Aβ clearance while simultaneously reducing hyperinsulinemia by drug or lifestyle interventions. Based on our findings, the pancreas may also serve as an important organ to study due to its inter-connectedness with liver function and its role in insulin synthesis as well as APP production. Lastly, future research should consider focusing on quantifying the contribution of periphery-born Aβ on AD outcomes. Overall, this review advocates for a shift towards greater emphasis on cardiometabolic health, cause-specific treatment, and combination therapies in the interest of offering the most promising interventions for AD.

## Author Contributions

MH and VB researched data and reviewed/edited manuscript. KA and DR researched data and reviewed manuscript. VH conceived the idea, contributed to discussion, researched data and reviewed/edited manuscript. All authors contributed to the article and approved the submitted version.

## Conflict of Interest

The authors declare that the research was conducted in the absence of any commercial or financial relationships that could be construed as a potential conflict of interest.

## Publisher’s Note

All claims expressed in this article are solely those of the authors and do not necessarily represent those of their affiliated organizations, or those of the publisher, the editors and the reviewers. Any product that may be evaluated in this article, or claim that may be made by its manufacturer, is not guaranteed or endorsed by the publisher.
